# A Case of a Giant Hemangioma of a Primary Cardiac Tumor

**DOI:** 10.7759/cureus.43818

**Published:** 2023-08-20

**Authors:** Tomohiro Nakajima, Tsuyoshi Shibata, Keishi Ogura, Yutaka Iba, Nobuyoshi Kawaharada

**Affiliations:** 1 Cardiovascular Surgery, Sapporo Medical University, Sapporo, JPN; 2 Division of Radiology and Nuclear Medicine, Sapporo Medical University, Sapporo, JPN

**Keywords:** targeted radiation therapy, benign cardiac tumor, giant tumor, hemangioma, cardiac tumor

## Abstract

We report a case of a 71-year-old female with a primary cardiac tumor. The patient had undergone surgery for uterine cancer 10 years ago and presented to a nearby clinic complaining of dyspnea on exertion. Chest X-ray revealed cardiac enlargement, prompting further investigations, which revealed a massive tumor protruding into the left atrium and extending toward the outer wall of the left ventricle. The patient was referred to a cardiac surgery department for myocardial biopsy. The tumor biopsy confirmed a diagnosis of a vascular tumor. Due to the tumor's large size and the difficulty in achieving complete resection, a conservative approach was chosen as the patient expressed a preference for non-surgical treatment. This is an extremely rare case of a large primary cardiac tumor, and we report it accordingly.

## Introduction

When investigating the frequency of tumors occurring in various organs throughout the body, it is found that the incidence of tumors originating from the heart is extremely rare, at less than 0.1%. Nevertheless, tumors can develop in the heart. Approximately 70% of the tumors that develop in the heart are considered benign, while malignant tumors account for about 30%.

Among benign tumors, mucous tumors are the most common, constituting about 50% of all benign tumors and approximately 30-40% of all heart tumors. Other benign tumors include lipomas, papillary elastomas, rhabdomyomas, fibromas, vascular tumors, and hamartomas. The decision to perform surgery for these benign tumors is based on the presence of symptoms and the risk of tumor embolism.

## Case presentation

**Figure 1:**
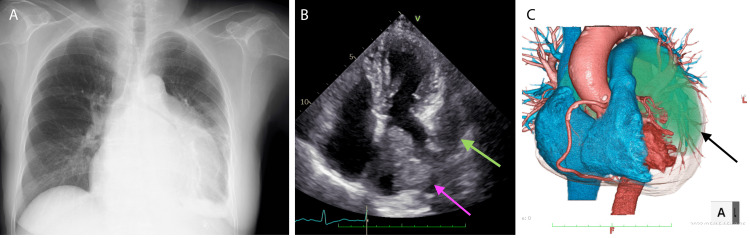
Preoperative findings. (A) Chest X-ray revealed expanding cardio-thoracic ratio. (B) Preoperative echocardiogram showed a cardiac tumor in the left atrium (pink arrow). The green arrows show the expanding tumor exceeding the left atrial wall. (C) Volume rendering computed tomography was shown. The green lesion was the cardiac tumor and was spreading to the left lateral wall (black arrow).

The patient is a 71-year-old female who underwent total hysterectomy through open abdominal surgery for cancer of the uterine body 10 years ago. She had no recurrence since then. She complained of dyspnea on exertion and underwent examination at a local clinic, where a chest X-ray revealed cardiac enlargement (Figure 1A). She was admitted to our cardiology department for further evaluation. Transthoracic echocardiography revealed a protruding tumor in the left atrium (Figure 1B), and a CT scan showed a tumor extending from the left atrium to the outer wall of the left ventricle (Figure 1C).

**Figure 2:**
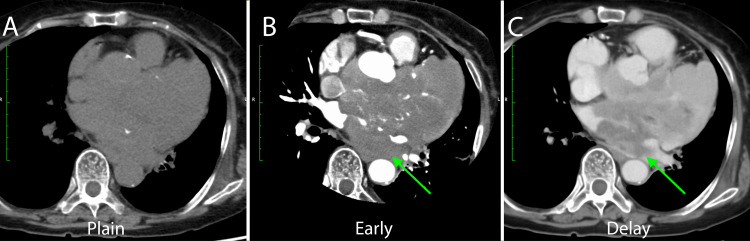
Findings on CT scans. (A) Plain CT. (B) Early-enhanced CT showed feeding arteries to the tumor (green arrow). (C) Delay enhanced indicated a low enhanced area of the cardiac tumor (green arrow). These features suggested that the tumor was rich in blood vessels.

As shown in Figure 2, early-phase contrast-enhanced images demonstrated tumor enhancement, and delayed-phase images showed further intense staining of the tumor. At this point, it was predicted to be a tumor with vascular proliferation.

**Figure 3:**
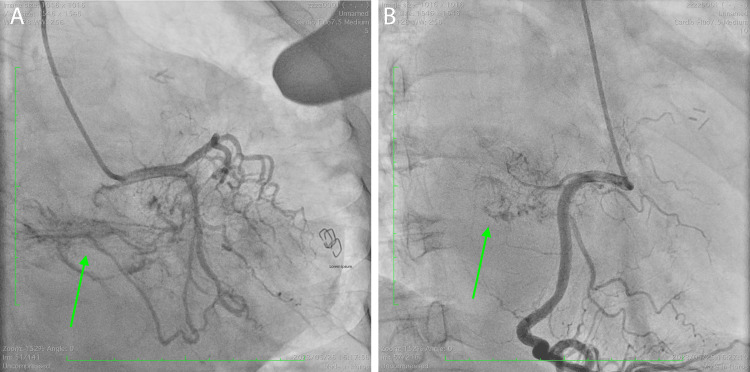
Coronary angiography. (A) Left coronary artery angiography. (B) Right coronary artery angiography. Both coronary arteries had feeding arteries to the cardiac tumor (green arrows).

Fluorodeoxyglucose-positron emission tomography (FDG-PET) computed tomography (CT) was performed, and although standardized uptake values (SUVs) were high in the cardiac tumor, no other sites showed elevated SUVs, leading to the diagnosis of a primary cardiac tumor. Coronary angiography revealed feeding vessels originating from the left circumflex artery and right coronary artery directed toward the tumor (Figure 3).

**Figure 4:**
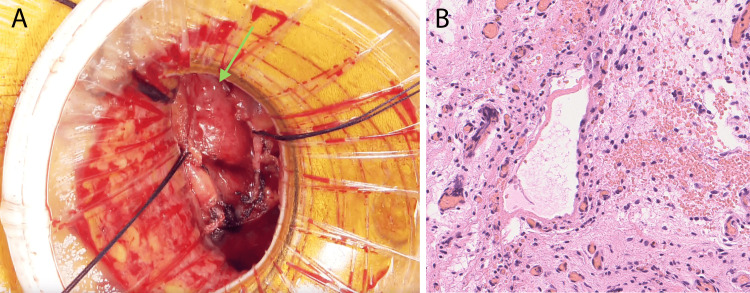
Operative findings. (A) Operative findings after pericardium cradle. The cardiac tumor was soft and full of vascular (green arrows). (B) Hematoxylin and eosin stain ×20 magnification. Pathological findings showed that it consisted of a dilated vascular lumen with a spongy appearance. The walls of the lumen were covered with irregularly proliferating smooth muscle.

Partial biopsy surgery was requested to confirm the tumor diagnosis. The surgery was performed under general anesthesia. A left fourth intercostal mini-thoracotomy was performed. The tumor did not extend beyond the epicardium. Upon opening the pericardium, only mild adhesions were observed in the pericardial cavity. The tumor was soft and yellowish (Figure 4A). It was excised as a 1 cm cubic specimen and submitted for pathological examination. The tumor was rich in blood vessels and prone to bleeding, consistent with the preoperative findings. The pathological diagnosis confirmed a benign vascular tumor (Figure 4B). Capillaries with poorly atypical endothelial cells are partially proliferating in a trabecular fashion.　

Treatment reports for cardiac vascular tumors have been previously documented, and based on those reports, treatment options were considered. For benign cardiac tumors, surgery should be considered when there are symptoms of heart failure or embolic symptoms caused by the tumor. In this case, heart failure symptoms were present, suggesting surgical indication. However, it was unclear whether the cause of heart failure was (a) reduced left atrial volume due to the occupation of the left atrium by the tumor, or (b) the tumor located outside the left ventricular lateral wall restricting its movement. Even if the tumor adhering to the left ventricular lateral wall was resected as much as possible, numerous feeding arteries from the coronary arteries were present. There was a high risk of artery damage leading to the coronary steal phenomenon, and it was considered that the risk of resection through surgery was high. We consulted the radiotherapy department to see if embolization of the nutrient vessels leading to the tumor was feasible, but they concluded that it would be difficult. Taking these findings into consideration, after discussing with the patient and her family, the decision was made to choose the best supportive care. The patient was discharged home 10 days after the surgical procedure.

## Discussion

Like other organs, tumors can also occur in the heart. In terms of frequency, they are rare, accounting for less than 0.1% of all autopsy cases. Among these cases, approximately 70% are benign tumors and 30% are malignant tumors [1]. The most common type of benign tumor is myxoma, which represents about half of all benign cardiac tumors and over 30% of all cardiac tumors. In addition to myxoma, there are other benign tumors such as lipomas, papillary elastofibromas, rhabdomyomas, fibromas, vascular tumors, atrioventricular nodal myxomas, and hamartomas [2]. Each of these tumors has distinct morphological features. Since these tumors are benign, they do not pose a direct threat to life. However, depending on the site of tumor occurrence, they can affect heart function or cause embolic events if parts of the tumor break off. Therefore, the general principle of treatment is to surgically remove the tumor.

In this case, a cardiac tumor was discovered as a result of heart failure. The biopsy results diagnosed it as a benign vascular tumor originating from the heart. Several case reports of excision of benign cardiac tumors have been reported previously [3,4]. However, those papers limited the tumors to specific areas. In this case, although the tumor itself was benign, it had widespread and compressed the outer wall of the left ventricle from within the left atrium. Contrast-enhanced CT and coronary angiography revealed the presence of multiple feeding arteries supplying the tumor. Considering it was a benign tumor, surgical excision was considered. However, complete removal of the tumor was not possible, and the effectiveness of partial excision in improving heart failure was uncertain. There were concerns about myocardial ischemia due to damage to the feeding artery or the main coronary artery during tumor excision.

In recent years, treatment methods involving embolization of feeding arteries from the coronary artery have been reported [5]. Consultation with a radiologist concluded that the treatment was not suitable due to the small diameter of the feeding artery. When these findings were conveyed to the patient and their family, the patient opted not to undergo invasive treatment and requested to observe the progress of the condition.

We experienced a case in which heart failure symptoms were caused by a giant vascular tumor of cardiac origin. Although it was a benign tumor and excision was considered, the tumor was too large to be completely removed. Furthermore, even if partial excision was performed, the frequency of complications was expected to outweigh the effectiveness of the procedure. Therefore, in this case, surgery was not performed.

## Conclusions

We encountered a case of a giant vascular tumor originating from the heart. A partial biopsy of the tumor was performed surgically, leading to the diagnosis of hemangioma. If the tumor is small, resection is often considered, but in this case, the tumor was so large that complete resection was not possible. After conducting an investigation, we considered various treatment options but ultimately decided to observe the patient's condition conservatively. 
